# Comparison of Hybrid Dynamic Stabilization with TLIF Versus Dynamic Stabilization Alone in Degenerative Lumbar Instability

**DOI:** 10.3390/diagnostics15151887

**Published:** 2025-07-28

**Authors:** Uzay Erdogan, Gurkan Berikol, Ibrahim Taha Albas, Mehmet Yigit Akgun, Tunc Oktenoglu, Ozkan Ates, Ali Fahir Ozer

**Affiliations:** 1 Department of Neurosurgery, Bakirkoy Prof. Dr. Mazhar Osman Training and Research Hospital for Psychiatry, Neurology and Neurosurgery, Istanbul 43606, Turkey; uzayerdogan@gmail.com; 2 Department of Neurosurgery, Health Sciences University, Sincan Training and Research Hospital, Ankara 06949, Turkey; dr.berikol@gmail.com; 3 Department of Neurosurgery, Istanbul Physical Therapy and Rehabilitation Training and Research Hospital, Istanbul 43606, Turkey; taha.albas@gmail.com; 4 Department of Neurosurgery, Koc University Hospital, Istanbul 43606, Turkey; tuncoktenoglu@gmail.com (T.O.); atesozkan@hotmail.com (O.A.); alifahirozer@gmail.com (A.F.O.); 5 Spine Center, Koc University Hospital, Istanbul 43606, Turkey; 6 Koc University Research Center for Translational Medicine, Biomechanics and Endurance Laboratory, Istanbul 43606, Turkey; 7 Bioengineering and Orthopaedic Surgery Colleges of Engineering and Medicine, University of Toledo, Toledo, OH 43606, USA

**Keywords:** lumbar fusion, dynamic stabilization, TLIF, imaging assessment, adjacent segment disease, hybrid constructs

## Abstract

**Objective**: This study aimed to compare the clinical and radiological outcomes of dynamic rod stabilization with and without transforaminal lumbar interbody fusion (TLIF) in patients undergoing surgery for degenerative lumbar instability. Specifically, we evaluated the prognostic value of hybrid systems in reducing adjacent segment disease (ASD), enhancing fusion rates, and improving functional outcomes. **Methods**: A retrospective analysis was conducted on 62 patients treated between 2019 and 2022. Group 1 (*n* = 34) underwent dynamic rod stabilization alone, while Group 2 (*n* = 28) received dynamic stabilization combined with TLIF. Radiological assessments included disk height index (DHI) and fusion rates. Clinical outcomes were measured using the Visual Analog Scale (VAS) for back and leg pain at baseline, 12, and 24 months. Statistical analysis was performed using Jamovi^®^ software (version 2.4.1). **Results**: The hybrid group (dynamic + TLIF) demonstrated significantly higher anterior fusion rates (*p* < 0.001) and greater improvement in VAS scores for back (*p* = 0.005) and leg pain (*p* < 0.001) at 12 months. Although operative time was longer (*p* = 0.002), there was no significant difference in hospital stay (*p* = 0.635). No significant differences were observed in ASD development (*p* = 0.11) or pseudoarthrosis (*p* = 0.396). The hybrid group maintained better lumbar lordosis and higher adjacent segment DHI. **Conclusions**: Hybrid dynamic stabilization combined with TLIF provides superior clinical outcomes and fusion rates compared to dynamic stabilization alone, without significantly increasing the risk of ASD. These findings support the use of hybrid constructs as a balanced strategy for treating degenerative lumbar instability.

## 1. Introduction

Lumbar fusion surgery has demonstrated high rates of radiographic success in treating degenerative lumbar instability. However, clinical outcomes and patient satisfaction often fall short of expectations. A meta-analysis evaluating the efficacy of lumbar spinal fusion reported a wide range of success rates, varying between 16% and 95%, with an average of 70% [1]. Despite the radiological success, rigid spinal instrumentation can increase mechanical stress on adjacent segments, contributing to adjacent segment degeneration (ASD) [2,3]. Furthermore, fusion surgery is associated with notable drawbacks, including prolonged operative time, increased risk of infection, donor site morbidity, implant fatigue failure, pseudoarthrosis, and persistent postoperative pain [4,5].

According to Wolff’s law, bone adapts its architecture in response to the mechanical loads it experiences, undergoing remodeling to better resist those forces. Adequate physiological loading is essential for bone graft incorporation and fusion maturation. In rigid fixation systems, however, the anterior column may be shielded from compressive forces due to vertical load transfer through stiff posterior instrumentation—a phenomenon known as stress shielding. This can impair osteointegration and fusion. In contrast, dynamic stabilization systems allow partial load sharing with the anterior spinal column, preserving compressive forces on the graft and potentially enhancing fusion rates through more physiological load transmission.

In rigid fixation systems, vertical load transmission can lead to “stress shielding,” in which the graft is inadequately loaded, impairing the fusion process [6]. In contrast, dynamic stabilization systems allow partial load sharing with the anterior spinal column, thereby maintaining compressive forces on the graft and potentially enhancing fusion rates [6,7].

The development of alternative spinal implants has focused on reducing ASD, minimizing implant-related complications, and preserving physiological spinal motion. Dynamic stabilization systems aim to limit abnormal segmental motion and promote balanced load distribution across spinal structures while still preserving partial mobility [8,9,10]. These systems adapt to postural changes and help maintain segmental alignment without the rigidity of traditional fusion constructs.

In response to the limitations of both rigid fusion and dynamic stabilization alone, hybrid constructs combining interbody fusion with dynamic posterior instrumentation have been proposed [11,12,13,14]. These systems aim to offer the mechanical benefits of fusion while mitigating the drawbacks of rigidity. In the present study, we evaluated the clinical and radiological outcomes of patients treated with dynamic stabilization alone versus those treated with a hybrid approach combining dynamic rods and transforaminal lumbar interbody fusion (TLIF). Our 2-year follow-up data are intended to contribute to evidence-based decision-making in the surgical management of degenerative lumbar spine disorders.

## 2. Materials and Methods

In this study, all procedures were performed in accordance with the ethical standards of the institutional and national research committee and with the 1964 Helsinki Declaration and its later amendments or comparable ethical standards. Informed consent was obtained from all participants included in the study.

### 2.1. Study Design and Patient Groups

A retrospective cohort study was conducted involving 62 patients who underwent lumbar spine surgery between 2019 and 2022 for degenerative instability at the L3–L5 levels. Patients were divided into two groups based on the surgical technique, as follows:

Group 1 (*n* = 34): Dynamic rod stabilization at L3–L5;

Group 2 (*n* = 28): TLIF at the L4–L5 level combined with dynamic rod stabilization at L3–L5.

Transforaminal lumbar interbody fusion (TLIF) was performed in patients with advanced disk degeneration (Pfirrmann grades 4–5) at the L4–L5 level, as determined by preoperative MRI. The groups were compared based on clinical and radiological outcomes over a 24-month follow-up period.

### 2.2. Surgical Procedure

In the hybrid group, surgery was performed using standard TLIF techniques following adequate neural decompression. A PEEK or helical rod-based dynamic stabilization system was applied posteriorly after pedicle screw placement.

In the dynamic-only group, decompression was followed by transpedicular screw fixation and dynamic rod stabilization without interbody fusion. In a small subset of patients undergoing rigid fusion, instrumentation included rigid rods with or without interbody cages, depending on intraoperative findings.

Pedicle screws were inserted under intraoperative fluoroscopic guidance. Postoperative imaging was reviewed to confirm proper screw placement. No cases of significant malposition or revision due to pedicle breach were encountered.

### 2.3. Radiological and Clinical Assessment

**Figure 1:**
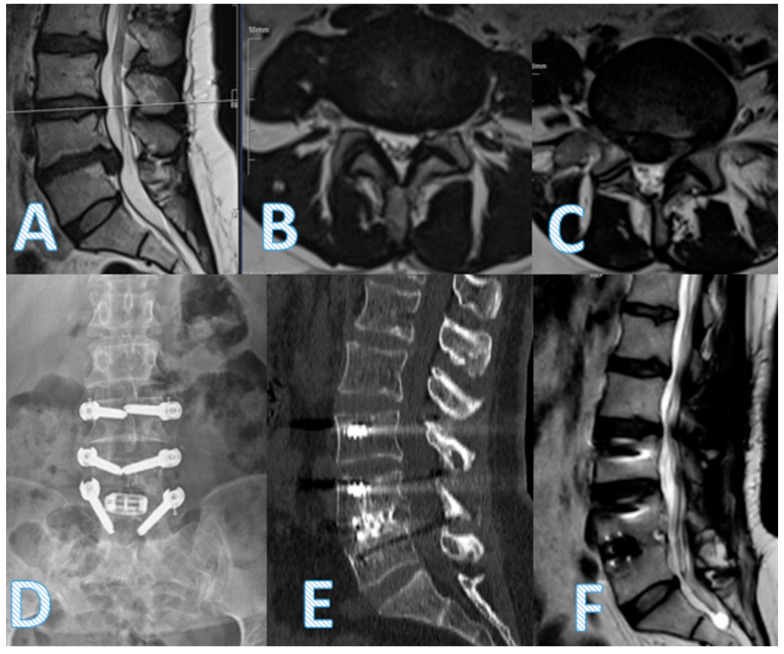
Preoperative sagittal (**A**) and axial MR images at the L3–4 (**B**) and L4–5 (**C**) levels demonstrate severe foraminal stenosis and compression of the exiting nerve roots at L4–5, more prominent on the right, consistent with the patient’s symptoms of neurogenic claudication and bilateral leg pain. Postoperative anteroposterior X-ray (**D**), sagittal CT (**E**), and sagittal MR (**F**) images show decompression and stabilization achieved through L4–5 transforaminal lumbar interbody fusion (TLIF) using a PEEK rod and interbody cage, with restored disk height and adequate neural decompression.

Radiological and clinical evaluations were performed preoperatively and postoperatively at 12 and 24 months. Imaging included standing anteroposterior and lateral radiographs of the lumbar spine at all time points. MRI was used preoperatively to assess the degree of disk degeneration (Figure 1).

Fusion was evaluated using sagittal CT imaging at 24 months in all patients to confirm anterior and posterior arthrodesis status.

Disk height indices (DHI) of the upper and lower adjacent segments were calculated from radiographs. To ensure reproducibility, DHI measurements were independently performed by two spine surgeons for a randomly selected subset of 15 patients. Interobserver and intraobserver reliability were assessed using intraclass correlation coefficients (ICC), yielding values of 0.91 and 0.94, respectively, indicating excellent agreement.

Clinical assessments included Visual Analog Scale (VAS) scores for both back and leg pain. Additional perioperative variables such as operative time, hospital stay, intraoperative blood loss, and complication rates were recorded.

### 2.4. Statistical Analysis

All statistical analyses were performed using Jamovi^®^ for Windows (version 2.4.11). Continuous variables were presented as means, standard deviations, medians, and standard errors. The Shapiro–Wilk test was used to assess normality of distribution. Comparisons between groups were made using the Mann–Whitney U test for non-normally distributed data and the independent *t*-test for normally distributed data. Categorical variables were analyzed using the chi-square or Fisher’s exact test, as appropriate. A *p*-value of <0.05 was considered statistically significant.

## 3. Results

The average age of patients in the dynamic-only group was significantly lower than that of the dynamic + TLIF group (*p* = 0.003) (Table 1). Operative time was significantly longer in the dynamic + TLIF group (*p* = 0.002); however, there was no statistically significant difference in hospital stay between the two groups (*p* = 0.635) (Table 2).

The operative time ranged from 150 to 420 min in the dynamic-only group and from 180 to 480 min in the dynamic + TLIF group, reflecting expected variability based on surgical complexity and level of involvement. No significant outliers were observed that would disproportionately influence group comparisons.

There were no statistically significant differences in lower segment disk height indices (DHI) between the groups at any time point, including preoperative, postoperative, 12-month, and 24-month evaluations (Table 3). However, upper segment DHI values were significantly lower in the dynamic-only group across follow-ups (Table 4). Despite this, no significant differences were found between the groups in terms of adjacent segment disease (ASD) development (*p* = 0.11), complications, or pseudoarthrosis (*p* = 0.396).

**Figure 2:**
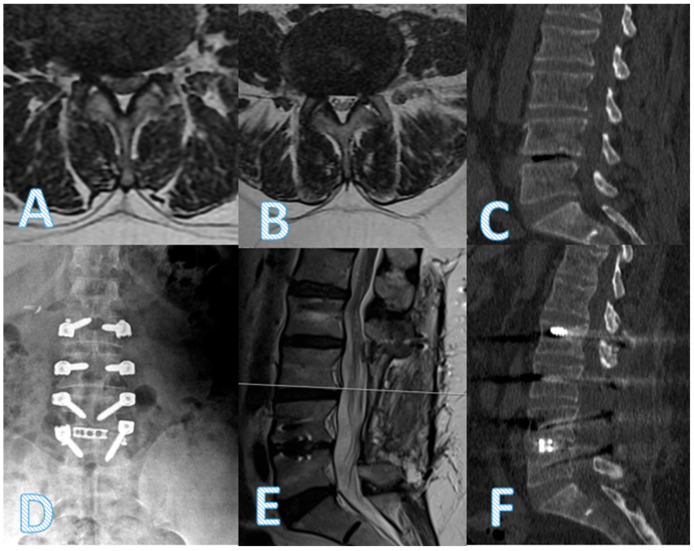
A 58-year-old female patient presented with low back pain (VAS 7) and right-dominant leg pain (VAS 8). Axial MR images at the L3–4 (**A**) and L4–5 (**B**) levels reveal bilateral foraminal stenosis, more severe at L4–5. Preoperative sagittal CT (**C**) shows disk space narrowing and endplate sclerosis at L4–5. Postoperative anteroposterior X-ray (**D**), sagittal T2-weighted MRI (**E**), and sagittal CT (**F**) demonstrate adequate decompression and stabilization following L4–5 transforaminal lumbar interbody fusion (TLIF) with a PEEK rod and interbody cage.

Anterior fusion development at 24 months was significantly higher in the dynamic + TLIF group (*p* < 0.001), although posterior fusion rates did not differ (*p* = 0.780). Radiological findings in representative patients illustrate successful decompression and implant positioning in the hybrid group (Figure 2).

**Figure 3:**
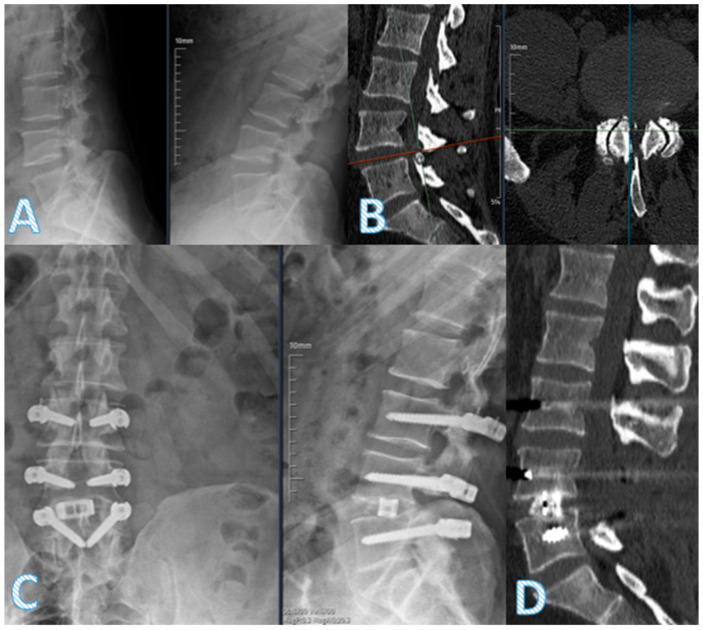
A 52-year-old female patient presented with severe low back pain (VAS 9), bilateral leg pain (VAS 7), neurogenic claudication at 50 m, and urge incontinence. Preoperative dynamic lateral lumbar X-rays in flexion and extension (**A**) and CT images (**B**) show advanced degeneration and instability at the L4–5 level with marked disk space narrowing. Postoperative anteroposterior and lateral lumbar radiographs (**C**) and sagittal CT (**D**) demonstrate successful decompression and stabilization following L4–5 transforaminal lumbar interbody fusion (TLIF) using a PEEK rod and interbody cage.

Both groups demonstrated statistically significant improvement in VAS scores for back and leg pain. However, the dynamic + TLIF group showed significantly greater improvements at the 12-month follow-up in both back (*p* = 0.005) and leg pain (*p* < 0.001) (Table 5). At 24 months, the hybrid group continued to show favorable clinical and radiological parameters, including anterior fusion and disk height maintenance. Pre- and postoperative imaging of a representative hybrid patient further demonstrates these outcomes (Figure 3), though it should be noted that operative time was longer in this group.

## 4. Discussion

**Figure 4:**
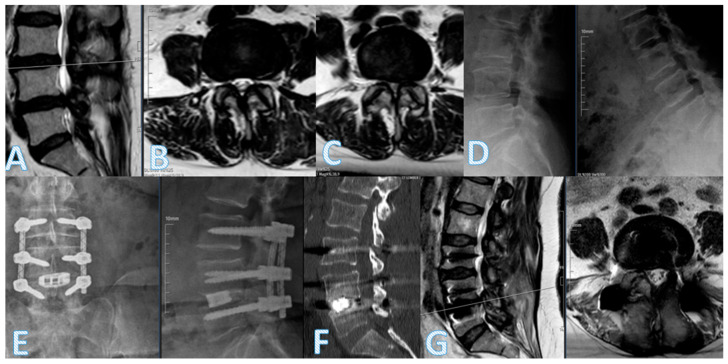
A 63-year-old female patient presented with severe low back pain (VAS 9) and left-dominant leg pain (VAS 8), accompanied by neurogenic claudication at 50 m. Preoperative sagittal T2-weighted MR (**A**), axial MR at L3–4 (**B**), and axial MR at L4–5 (**C**) demonstrate advanced disk degeneration and bilateral foraminal stenosis, most prominent at the L4–5 level. Dynamic flexion-extension lateral lumbar X-rays (**D**) show segmental instability. Postoperative anteroposterior and lateral radiographs (**E**), sagittal CT (**F**), and sagittal T2-weighted MR (**G**) confirm successful L4–5 transforaminal lumbar interbody fusion (TLIF) with a dynamic rod and interbody cage, showing restoration of disk height, decompression of neural structures, and stable implant positioning.

Hybrid stabilization systems aim to combine the mechanical advantages of interbody fusion—such as effective neural decompression and restoration of disk height—with the motion-preserving benefits of dynamic posterior instrumentation. While rigid fusion techniques provide immediate segmental stability and promote fusion, they are associated with an increased risk of adjacent segment disease (ASD), reported to occur in 23% to 43% of cases following posterior fusion [1,2,3,15]. By contrast, hybrid constructs are designed to mitigate these complications by offering a biomechanical transition between fused and mobile segments (Figure 4).

Several biomechanical and clinical studies have suggested that dynamic or hybrid instrumentation may reduce excessive motion at adjacent levels, thereby delaying the onset of ASD, although not necessarily preventing it entirely [6,9,13]. In our study, patients treated with dynamic stabilization alone were compared to those who underwent TLIF in combination with dynamic rods. Notably, both groups consisted of relatively young patients (under 60 years) and underwent surgery across a maximum of three levels—factors known to be associated with a lower incidence of ASD [16,17].

The TLIF group demonstrated significantly higher anterior fusion rates and better preservation of lumbar lordosis, both of which may contribute to improved sagittal alignment and long-term stability. Importantly, despite longer operative times in the TLIF group, there was no significant increase in complication rates or hospital stay, indicating that the added surgical complexity did not adversely affect short-term recovery.

Clinical outcomes, as assessed by VAS scores for both back and leg pain, improved significantly in both groups. However, the hybrid group exhibited superior improvements at the 12-month follow-up, consistent with previous literature suggesting that anterior fusion may enhance early pain relief and functional outcomes [11,18,19]. Although 1-month scores favored the TLIF group, no significant difference persisted at the 24-month evaluation, suggesting that both strategies offer sustained symptom relief over time. It is worth noting that the minimal clinically important difference (MCID) for VAS in spinal surgery is typically defined as 1.5 to 2.0 points. The observed improvements in both groups exceeded these thresholds, indicating clinically meaningful pain relief.

Biomechanical literature supports the concept that dynamic stabilization may promote more physiological load sharing and help distribute mechanical stress across spinal segments [6,7]. Hybrid constructs, by integrating dynamic posterior support with rigid anterior fixation, may buffer against abrupt mechanical transitions and minimize adjacent segment overload. Additionally, TLIF contributes to sagittal correction, which is a crucial factor in reducing upper segment degeneration [12,14,20,21].

Although pseudoarthrosis rates were evaluated radiologically and showed no significant difference between groups, other potential complications, such as perineural adhesions or facet joint overload, were not systematically assessed and were not clinically evident within the study period. Given that such complications are often reported in the literature, particularly with long-term follow-up, our findings may not fully capture the extended risk profile associated with dynamic or hybrid stabilization.

In this study, fusion was applied selectively to the most degenerated distal segment (L4–L5), while dynamic stabilization was applied at adjacent levels to reduce stress transmission. This targeted approach may help preserve motion while protecting adjacent segments and facet joints.

Although no significant differences in ASD development were observed between groups at the 24-month follow-up, it is important to acknowledge that ASD often manifests years after surgery. As such, our findings may not fully reflect the long-term incidence of adjacent segment pathology. Extended follow-up periods will be essential in future studies to determine the sustained efficacy of hybrid stabilization strategies in preventing late-onset ASD.

The observed age difference between groups—though statistically significant—was relatively modest and may not have had a substantial impact on the main clinical trends. Nevertheless, age-related variation in degenerative progression and pain tolerance warrants cautious interpretation of between-group comparisons.

Given the retrospective design and lack of randomization, the possibility of unmeasured confounding influencing treatment allocation and outcomes cannot be excluded. Therefore, the comparative findings between the hybrid and dynamic-only groups should be interpreted with caution, and future randomized controlled trials are warranted to validate these results.

Given the number of outcome variables assessed at multiple time points, there is an inherent risk of Type I error due to multiple comparisons. While no formal correction method (e.g., Benjamini–Hochberg) was applied due to the study’s limited sample size, we acknowledge that some statistically significant findings may reflect chance associations. Future studies with larger cohorts should incorporate multiplicity adjustment to enhance statistical validity.

## 5. Clinical Implications

The growing body of evidence supporting dynamic stabilization systems has contributed to evolving perspectives on lumbar spine surgery. Previous studies have demonstrated that dynamic stabilization can be safely and effectively applied in cases of disk herniation and unilateral spinal pathologies, with favorable clinical outcomes and low complication rates [22,23]. In particular, dynamic instrumentation has been shown to preserve motion, reduce adjacent segment stress, and provide long-term stability in selected patient populations. Moreover, in the context of complex deformities, dynamic stabilization has emerged as a potential alternative to rigid fusion, particularly when tailored according to deformity classification systems such as Silva-Lenke and Berjano-Lamartina [24,25]. The present study builds upon these findings by evaluating a hybrid approach that combines dynamic posterior instrumentation with interbody fusion, aiming to balance segmental stability with motion preservation. Our results further support the feasibility of dynamic systems not only as stand-alone options but also as valuable components in hybrid constructs for degenerative lumbar instability.

## 6. Future Directions

Future prospective studies with larger patient cohorts and longer follow-up durations are essential to confirm the advantages of hybrid stabilization systems. Randomized controlled trials comparing hybrid constructs with conventional rigid fusion or total dynamic stabilization are particularly needed. In addition, the incorporation of functional outcome measures and patient-reported satisfaction scores would further validate the clinical impact of hybrid approaches. Biomechanical studies evaluating load transmission and facet joint stress in hybrid configurations could also help optimize implant design and segment selection.

## 7. Limitations

Despite the valuable insights provided by this study, several limitations should be noted. First, the retrospective design introduces inherent risks of selection bias and limits the ability to establish causal relationships. Second, the relatively small sample size may reduce the statistical power to detect differences in certain subgroups or outcome measures. Third, while the follow-up period of 24 months allows for short- to mid-term evaluation, longer-term follow-up is necessary to assess the durability of fusion, risk of adjacent segment disease (ASD), and implant performance over time. Fourth, the study did not include functional outcome scales beyond VAS, such as ODI or SF-36, which could have provided a more comprehensive assessment of patient quality of life. Fifth, the baseline age imbalance between groups, with the hybrid group being significantly older on average. This may have influenced pain perception, degeneration rates, or fusion capacity. Although both groups showed comparable improvements over time, age-related factors should be considered when interpreting the outcomes. Sixth, the absence of randomization and the inability to perform multivariable adjustment or propensity score matching limit our ability to fully account for potential confounding variables. As such, unmeasured factors may have influenced treatment selection and outcomes. Lastly, although outcomes were recorded at multiple time points, the use of repeated univariate comparisons instead of a mixed-effects model limits the ability to fully account for within-subject correlation and treatment-by-time interactions. Future prospective analyses should consider longitudinal modeling to address this limitation.

## 8. Conclusions

This study demonstrates that hybrid stabilization systems combining TLIF with dynamic posterior instrumentation offer a balanced and effective strategy for the management of degenerative lumbar instability. By leveraging the decompression and fusion benefits of TLIF while preserving segmental motion with dynamic rods, these constructs may reduce the risk of adjacent segment degeneration and implant-related complications. The observed improvements in fusion rates, radiological parameters, and clinical outcomes support the use of hybrid systems as a viable alternative to rigid fixation, particularly in select patient populations. Larger, prospective studies with long-term follow-up are warranted to validate the durability and biomechanical advantages of this approach.

## Figures and Tables

**Table 1:** Age status between groups.

	Group	*N*	Mean	Median	SD	SE
**Age**	1	34	50.3	53.0	8.08	1.39
	2	28	56.7	56.0	8.01	1.51

**Table 2:** Operation time and hospital stay.

	Group	*N*	Mean	Median	SD	SE
Operation time	1	34	274.18	245.00	102.00	17.493
	2	28	316.32	315.00	82.31	15.555
Hospital stay	1	34	3.71	4.00	1.19	0.205
	2	28	4.07	3.00	2.31	0.436

**Table 3:** Adjacent lower segment disk height indices.

Lower Segment Disk Height Indices (dhi)	Group	*N*	Mean	Median	SD	SE
Lower segment dhi preop	1	34	0.363	0.365	0.0895	0.0153
	2	28	0.345	0.333	0.0703	0.01329
Lower segment dhi postop	1	34	0.380	0.365	0.0917	0.0157
	2	28	0.347	0.338	0.0464	0.00876
Lower segment dhi 12th month	1	34	0.372	0.342	0.0953	0.0163
	2	28	0.346	0.344	0.0485	0.00916
Lower segment dhi 24th month	1	34	0.353	0.337	0.0949	0.0163
	2	28	0.345	0.350	0.0579	0.01093

**Table 4:** Adjacent upper segment disk height indices.

Upper Segment Disk Height Indices	Group	*N*	Mean	Median	SD	SE
Upper segment dhi preop	1	34	0.253	0.246	0.1023	0.0175
	2	28	0.279	0.288	0.0522	0.00986
Upper segment dhi postop	1	34	0.265	0.259	0.0972	0.0167
	2	28	0.293	0.302	0.0561	0.01060
Upper segment dhi 12th month	1	34	0.248	0.261	0.0922	0.0158
	2	28	0.293	0.297	0.0694	0.01311
Upper segment dhi 24th month	1	34	0.239	0.245	0.0992	0.0170
	2	28	0.289	0.307	0.0751	0.01420

**Table 5:** Comparison of back and leg VAS values.

	Group	*N*	Mean	Median	SD	SE
Back VAS preop	1	34	8.74	9.00	1.163	0.199
	2	28	8.46	8.00	1.036	0.196
Back VAS postop	1	34	6.15	6.00	1.329	0.228
	2	28	5.14	6.00	2.103	0.397
Back VAS 12th month	1	34	4.59	4.00	1.209	0.207
	2	28	3.68	4.00	1.188	0.225
Back VAS 24th month	1	34	3.00	2.50	1.842	0.316
	2	28	2.68	3.00	1.416	0.268
Leg VAS preop	1	34	6.38	6.00	1.633	0.280
	2	28	6.96	7.00	1.551	0.293
Leg VAS postop	1	34	3.59	4.00	1.351	0.232
	2	28	3.00	3.00	1.440	0.272
Leg VAS 12th month	1	34	2.85	3.00	0.925	0.159
	2	28	2.21	2.00	0.568	0.107
Leg VAS 24th month	1	34	1.74	2.00	1.082	0.186
	2	28	1.32	1.00	1.056	0.200

## Data Availability

The datasets used and/or analyzed during the current study are available from the corresponding author upon reasonable request.
